# Feasibility of Using Electrocochleography for Objective Estimation of Electro-Acoustic Interactions in Cochlear Implant Recipients with Residual Hearing

**DOI:** 10.3389/fnins.2017.00337

**Published:** 2017-06-15

**Authors:** Kanthaiah Koka, Leonid M. Litvak

**Affiliations:** Research and Technology, Advanced Bionics LLCValencia, CA, United States

**Keywords:** residual hearing, cochlear implant, electrocochleography, ECochG, electro-acoustic stimulation, EAS and electro-acoustic interaction

## Abstract

Although cochlear implants (CI) traditionally have been used to treat individuals with bilateral profound sensorineural hearing loss, a recent trend is to implant individuals with residual low-frequency hearing. Patients who retain some residual acoustic hearing after surgery often can benefit from electro-acoustic stimulation (EAS) technologies, which combine conventional acoustic amplification with electrical stimulation. However, interactions between acoustic and electrical stimulation may affect outcomes adversely and are time-consuming and difficult to assess behaviorally. This study demonstrated the feasibility of using the Advanced Bionics HiRes90K Advantage implant electronics and HiFocus Mid Scala/1j electrode to measure electrocochleography (ECochG) responses in the presence of electrical stimulation to provide an objective estimate of peripheral physiologic EAS interactions. In general, electrical stimulation reduced ECochG response amplitudes to acoustic stimulation. The degree of peripheral EAS interaction varied as a function of acoustic pure tone frequency and the intra-cochlear location of the electrically stimulated electrode. Further development of this technique may serve to guide and optimize clinical EAS system fittings in the future.

## Introduction

Because of advances in electrode array technology and surgical technique, patients with low-frequency residual acoustic hearing could benefit from cochlear implants (CI) (Balkany et al., 2006; Fraysse et al., 2006). Although some of these individuals lose their residual hearing completely after implant surgery, others can experience partial or full retention of their acoustic hearing (Radeloff et al., 2012; Dalbert et al., 2015). Subjects with residual hearing often can benefit from electro-acoustic stimulation (EAS) technologies, which combine conventional acoustic amplification with electrical stimulation (Von Ilberg et al., 1999; Turner et al., 2008).

One of the challenges in optimizing EAS benefit in individual patients is understanding the interactions between acoustic and electrical hearing. Psychometric studies indicate that acoustic thresholds can be increased in the presence of electrical stimulation, thereby suggesting peripheral electro-acoustic interactions (Lin et al., 2011). Systematic programming modifications such as switching off electrodes or using overlapping or non-overlapping cross-over frequencies also can characterize electro-acoustic interactions and suggest ways to improve benefit (Polak et al., 2010; Karsten et al., 2013). The drawback to these behavioral techniques is that they are subjective and require too much time, thereby making them impractical for clinical use.

Consequently, it would be valuable to take advantage of objective responses to help clinicians program EAS devices optimally. The electrically evoked compound action potential (ECAP) is a physiologic response that reflects auditory nerve activity and can serve as an objective measure of electro-acoustic interactions in the same ear (Abbas et al., 2002; Stronks et al., 2010, 2012). For example, Abbas et al. (2002) showed electro-acoustic interactions in cats with residual hearing. They observed secondary peaks in ECAP amplitudes and hypothesized that these peaks resulted from electrical stimulation of hair cells, often referred to as electrophonics. They also showed a decrease in ECAP amplitude in the presence of wide-band acoustic noise, thus indicating the presence of peripheral electro-acoustic interactions. Similarly, Stronks et al. (2012) observed a decrease in ECAP amplitude in the presence of broadband noise in guinea pigs.

Electrocochleography (ECochG) is a procedure that offers potential for assessing peripheral electro-acoustic interactions objectively. The ECochG response is comprised of electrical potentials generated by the hair cells and auditory nerve. The cochlear microphonic (CM) represents the combination of transducer currents primarily through the outer hair cell stereocilia (Dallos, 1973) and is known to follow the fine structure of the stimulus waveform. The auditory nerve neurophonic (ANN) is assumed to reflect the phase-locking activity of the auditory nerve fibers (Snyder and Schreiner, 1984; Lichtenhan et al., 2013; Fitzpatrick et al., 2014; Forgues et al., 2014). The compound action potential (CAP) is generated by the auditory nerve in response to the onset and offset of the acoustic stimulus, and the summating potential (SP) is the direct current part of the response with multiple generators.

To date, the ability to measure ECochG responses in the presence of electrical stimulation in CI recipients has been limited by CI hardware capability due to stimulus artifacts. However, the back-telemetry capability and fast-recovery amplifier in the Advanced Bionics (AB) HiRes90K® cochlear implant offers the opportunity to measure ECochG responses reliably and to explore the feasibility of using ECochG to assess peripheral electro-acoustic interactions. the AB device can record ECochG responses to low frequency pure tones. By calculating the *Difference response*, that is, the difference between responses to alternating stimulus polarities, the odd harmonics of the tone frequency are emphasized. This calculation reflects the components of the response that follow stimulus periodicity. This *Difference response* is dominated by the CM, but also includes the largest part of the ANN (Forgues et al., 2014). In contrast, by calculating the *Summation response*, that is, the sum of the responses to alternating stimulus polarities, the even harmonics of the tone frequency are emphasized. This calculation includes components of the response that do not change with stimulus phase and thus reflects asymmetric distortions in the CM and ANN. Because these distortions are greater in the ANN than the CM, the ongoing component of the *Summation response* can be dominated by the ANN, when it is present. However, this part of the ANN is only the distortions, and so is smaller than the part that appears in the difference response.

This study explored the feasibility of using ECochG to assess electro-acoustic interactions objectively in implanted subjects with residual hearing in the presence of electrical stimulation. The study focused particularly on using the fast-recovery amplifier in the AB HiRes90K® cochlear implant to measure ECochG responses. The objective of the study was to show that it is feasible to record the *Difference response* and the *Summation response* in the presence of electrical stimulus artifacts. These measurements then would provide a way to objectively estimate electro-acoustic interactions. A hypothesis that these objective electro-acoustic interactions correlate with behaviorally measured electro-acoustic interactions was tested.

## Methods

Two methods were used to explore the interaction between acoustic and electrical stimulation in CI recipients with residual hearing. Experiment 1 evaluated the feasibility of recording acoustic ECochG responses in the presence of electrical stimulation. Those responses then were used to estimate electro-acoustic interactions objectively. Experiment 2 assessed electro-acoustic interactions behaviorally by measuring changes in acoustic thresholds in presence of electrical stimulation. These behavioral interactions then were compared with the objective electro-acoustic interactions from Experiment 1.

### Experiment 1

#### Objective

The aim of this experiment was to show the feasibility of recording acoustic ECochG responses in the presence of electrical stimulation. The *Difference response* amplitudes observed in presence of electrical stimulation were compared to baseline responses measured with no electrical stimulation to provide an objective estimation of electro-acoustic interactions.

#### Subjects

**Figure 1:**
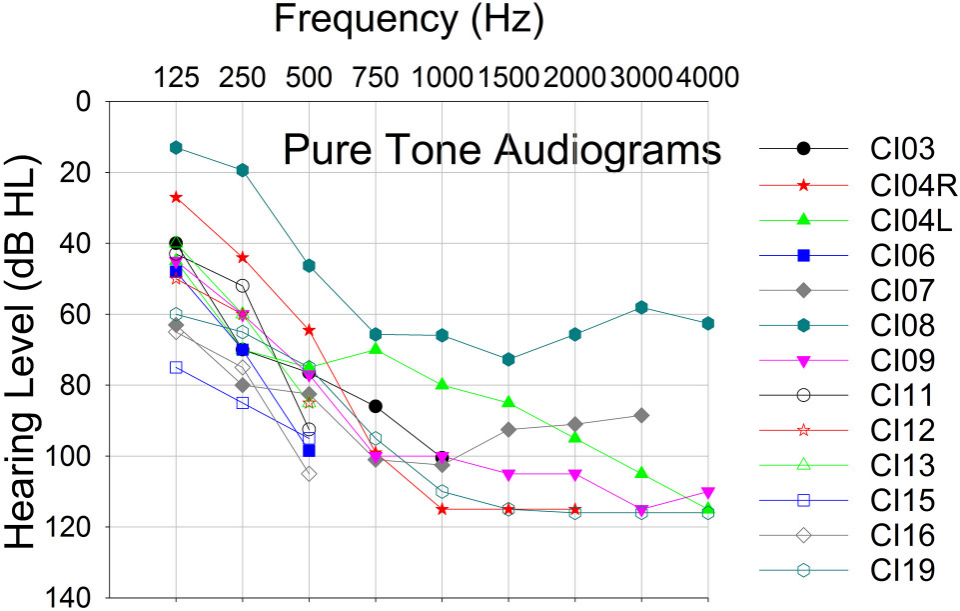
Pure tone audiograms for 12 study participants.

Twelve CI recipients with Advanced Bionics HiRes90K® cochlear implants and HiFocus MidScala® and 1J electrode arrays participated in this phase of the study. Eleven subjects were unilaterally implanted and one subject was a bilateral implant user, thereby yielding a total of 13 experimental ears. Table 1 shows the subjects' implant devices, duration of implant use and experimental participation. Figure 1 shows the pure-tone audiograms for these subjects, who exhibited different degrees of residual hearing. The etiology of the hearing loss is unknown for the group. All subjects provided written informed consent prior to participation. The study protocol (#20121035) was approved by the Western Institutional Review Board (WIRB).

**Table 1:** **Subject demographics**.

**Subject ID**	**Implant Type**	**Electrode Type**	**Implant usage (yrs)**	**Exp 1**	**Exp 2**
CI03	HiRes90K Advantage	MidScala	0.25	yes	yes
CI04L	HiRes90K Advantage	MidScala	1	yes	yes
CI04R	HiRes90K Advantage	MidScala	2	yes	yes
CI06	HiRes90K Advantage	MidScala	0.5	yes	yes
CI07	HiRes90K Advantage	MidScala	2	yes	no
CI08	HiRes90K Advantage	MidScala	1.67	yes	yes
CI09	HiRes90K Advantage	MidScala	1.5	yes	no
CI11	HiRes90K	HiFocus 1J	3	yes	no
CI12	HiRes90K Advantage	MidScala	1.5	yes	no
CI13	HiRes90K Advantage	MidScala	0.5	yes	yes
CI15	HiRes90K Advantage	MidScala	0.5	yes	yes
CI16	HiRes90K Advantage	MidScala	2	yes	no
CI19	HiRes90K Advantage	MidScala	1.5	yes	no

#### Equipment

The stimulus delivery and measurement system for assessing ECochG responses was like that described in Koka et al. (2016). The Advanced Bionics' Bionic Ear Data Collection System (BEDCS) research software was used to control stimulus delivery and ECochG response measurement. The acoustic stimuli were generated by an NI DAQ system (NI DAQ 6216, National Instruments Corporation, Austin, TX, USA) along with an audio amplifier (Sony PHA-2, Sony Corporation, New York, NY, USA) and presented through ER-3A insert earphones (Etymotic Research, Inc. Elk Grove Village, IL, USA). An ER-7 (Etymotic Research, Inc. Elk Grove Village, IL, USA) probe MIC was used to monitor the stimulus level in the ear canal. ECochG responses were measured using an Advanced Bionics' Clinical Programming Interface (CPI-II), Platinum Series Sound Processor (PSP), and Universal Headpiece (HP). The CPI-II delivered an external trigger to synchronize acoustic stimulus generation and response measurement through the implant. Frequencies including 125, 250, 500, 750, 1,000, and 2,000 Hz were studied. The stimulus delivery system had maximum levels of 90, 100, 105, 110, 110, 110 dB HL for those audiometric frequencies.

#### Stimulation and recording parameters

The acoustic stimulus for ECochG recording consisted of 50-ms tone bursts with a ramp duration of 5 ms (Hanning window) presented at each subject's most comfortable level (MCL) or at maximum stimulus level generated by test system at test frequency. ECochG responses were recorded using 240 presentations with alternating polarity (120 rarefaction and 120 condensation). From the responses to alternating polarities, the *Difference response* (difference between responses to the two polarities) or the *Summation response* (sum of responses to the two polarities) was computed.

The electrical stimulus consisted of a 50-ms biphasic pulse train with a phase duration of 36 μS. The inter-pulse gap was varied to produce pulse rates that ranged between 400 and 1,200 pulses per second (pps). The pulse trains were delivered at each subject's MCL. Electrical stimuli were delivered to either electrode 2 or electrode 3 in a monopolar manner using the case ground as the return electrode. Electrode 1 was used as the recording electrode. In some cases, electrode 2 was used as the recording electrode, and then either electrode 1 or 3 was used for stimulation. In the AB system, electrode 1 is the most apical electrode.

For recording, the ring electrode, located on the electrode lead outside of the cochlea, served as the reference electrode for the differential recording amplifier. The amplifier on the HiRes90K® Advantage implant was configured to have a gain of 1,000. Data were sampled at a rate of 9,280 sample/s, thus supporting a fast Fourier transform (FFT) up to 4,000 Hz. The response amplitudes were estimated as the peak value at stimulus frequency in the FFT spectrum. With these settings, the AB implant offers a relatively long recording window of 54.4 ms that can record ECochG waveforms for low-frequency stimuli down to 125 Hz.

#### Procedures

**Figure 2:**
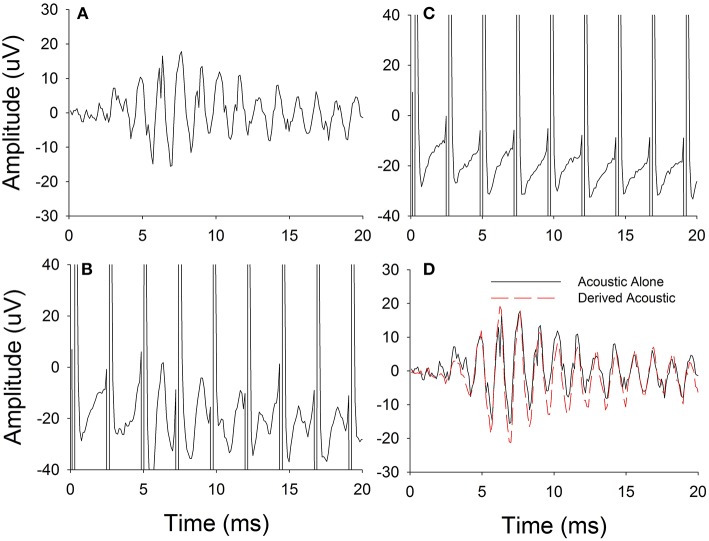
Experimental procedure used for estimating physiological electro-acoustic interactions for simultaneous stimulations. **(A)** *Difference responses* measured for acoustic stimulus alone. **(B)** *Difference responses* measured for electric and acoustic stimulus together. **(C)** *Difference responses* measured for electric stimulus alone. **(D)** *Derived acoustic* responses estimated by subtracting electric stimulus alone responses from responses to electric and acoustic stimuli presented together.

The procedure used for electro-acoustic interaction was simultaneous presentation of electric and acoustic stimuli. The electrical pulse rates and acoustic frequencies were kept disparate so that the acoustic responses could be differentiated from electrical stimulus artifacts in the FFT spectrum. Figure 2 illustrates the procedures used in this experiment. First, ECochG responses were recorded for the pure-tone acoustic stimulus presented alone (Figure 2A). Then the ECochG responses were recorded for the acoustic pure-tone stimulus and electrical pulse train presented simultaneously (Figure 2B). Following, ECochG responses were measured for the electrical pulse train alone (Figure 2C). Note that the responses in Figures 2B,C both show large stimulus artifacts during the electrical pulses, but the response to the acoustic stimulation still can be seen in Figure 2B, where the acoustic and electrical stimulation are presented together.

The electric-only responses were subtracted from electro-acoustic responses. This subtracted response was defined as the *Derived acoustic* response (Figure 2D). Finally, the *acoustic-alone* (Figure 2A) and *Derived acoustic* response (Figure 2D) amplitudes in the frequency spectrum were compared at the stimulus frequency to determine if any electrical-acoustic interaction was present. Even though not shown in Figure 2, a similar computational technique was used to calculate and analyze the interactions in the ANN.

Different electrodes were used for electrical stimulation and recording of ECochG responses to minimize stimulus artifact contamination of the recordings. The fast-recovery property of the evoked potential recording amplifier designed into the HiRes90K® Advantage cochlear implant allowed the amplifier, when it encountered large saturating stimulus artifacts, to quickly return from saturation into linear operation. This capability permitted recording of responses immediately after the stimulus artifact ended. Thus, electrical pulse rates closer to clinical stimulation rates could be explored to determine the feasibility of using this ECochG technique to complement everyday clinical programming.

### Experiment 2

#### Objective

The aim of this experiment was to estimate electro-acoustic interactions using a behavioral masking technique, i.e., the elevation of acoustic thresholds in the presence of an electrical stimulus masker. These behavioral electro-acoustic interactions were compared with the objective electro-acoustic interactions estimated in Experiment 1.

#### Subjects

A subset of the 6 subjects who participated in Experiment 1 took part in this phase of the study. Five were unilaterally implanted and one had two devices, resulting in a total of seven experimental ears. Table 1 indicates the six individuals composing this subset of subjects.

#### Stimulation and recording parameters

The experiment was conducted in a quiet room. If required, a foam plug was introduced in the contralateral ear to avoid distraction. The acoustic probe stimuli consisted of tone bursts at 125, 250, 500, 750, 1,000, and 2,000 Hz. The tone duration was 200 ms with 10-ms on/off ramps.

The electrical masker consisted of 500-ms pulse train of (cathodic first) biphasic pulses with phase durations of approximately 36 μs. The pulse rate was kept constant at 421 pps. Electrical stimulation was delivered at the same MCLs used in Experiment 1. When the probe and masker were delivered simultaneously, the acoustic tone burst was centered temporally within the electrical pulse train. The experimental design was similar to Lin et al. (2011).

#### Procedure

Unmasked and masked acoustic thresholds were measured using a three-interval, forced-choice procedure with a three-down-one-up search rule. Initially within a run, the acoustic stimulus levels were varied in 8-dB steps. After three reversals, the step size was reduced to 2 dB. Thresholds were calculated by averaging six reversals with a step size of 2 dB. Thresholds were measured for each acoustic stimulus presented alone and with the electrical masker. The threshold track was aborted if the acoustic signal level exceeded the maximum stimulation limit. Any changes in acoustic thresholds in the presence of electrical stimulation from the unmasked condition were evidence of electro-acoustic interactions.

## Results

### Feasibility of recording ECochG responses in the presence of electrical stimulation (Experiment 1)

**Figure 3:**
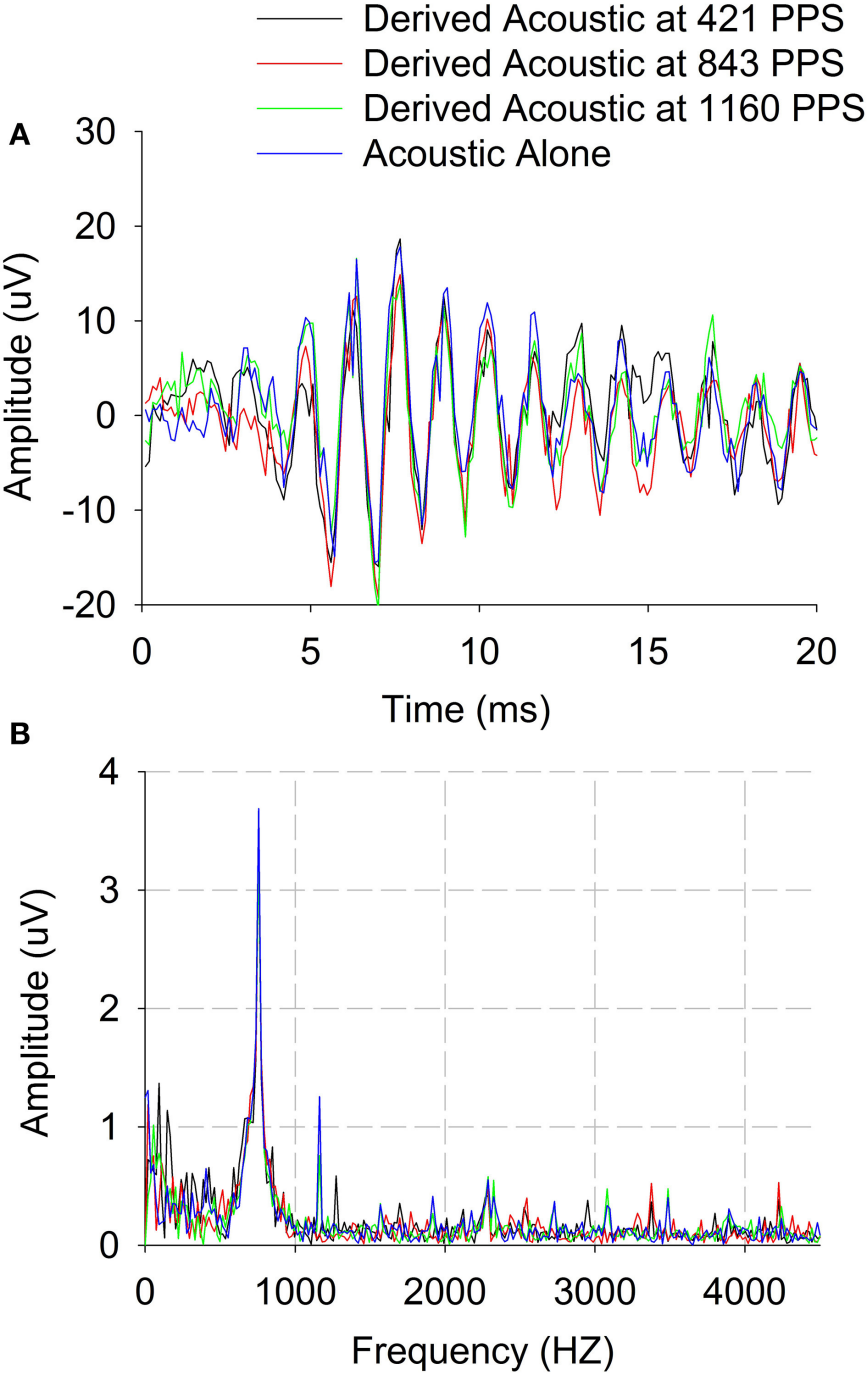
*Derived acoustic* and *Acoustic alone responses* of *Difference responses* (difference of alternating polarities) for three three electrical stimulation rates. **(A)** Time domain. **(B)** Frequency domain.

Figure 2 has shown feasibility of recording ECochG responses for an acoustic tone of 750 Hz and electrical stimulation rate of 421 pps. Then ECochG responses were also recorded for different electrical stimulation rates and *Derived acoustic responses* were estimated based on the technique described in Methods for Experiment 1. The peak amplitude of the *Difference response* to acoustic pure tones was assessed as a function of electrical pulse rate (400–1,200 pps). Figure 3 shows an example of the effect of stimulation rate on the *Derived acoustic* response for a 750-Hz pure-tone stimulus (CI08). Figure 3A overlays the time domain responses to the acoustic stimulus alone with *Derived acoustic* responses for electrical stimulation delivered at 421, 843, and 1,160 pps. Figure 3B shows the same four responses in the frequency domain.

The time domain data show no visible residual stimulus artifacts after template subtraction. The frequency spectra show some stimulus artifacts around 1,160 Hz which appear to be harmonic or at the electrical stimulation rate. Nonetheless, these stimulus artifacts were clearly different from the *Difference response* at 750 Hz. In this example, there is no evidence of peripheral electro-acoustic interactions as indicated by no differences in the waveforms or spectra for the *Derived acoustic* responses compared to the *acoustic-alone* responses. These results demonstrate the feasibility of recording acoustic responses in the presence of electrical stimuli delivered at different stimulation rates.

### Objective estimation of electro-acoustic interactions through ECochG responses (Experiment 1)

**Figure 4:**
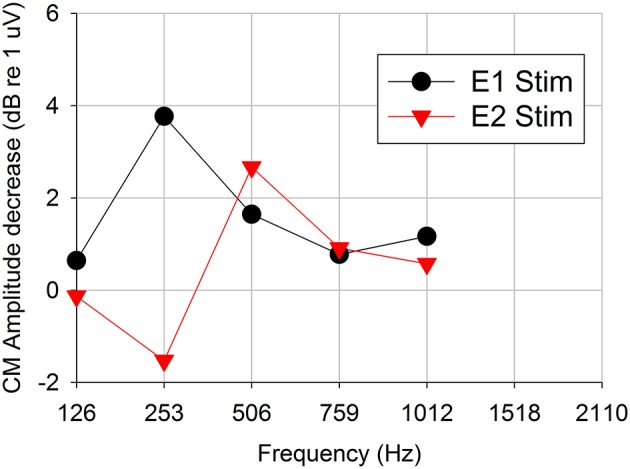
*Difference response* amplitude changes as a function of acoustic stimulation frequency for 421-pps electrical stimulation on two two different electrodes (representative subject CI04L). Positive dB-values indicate decreases (suppression) in the acoustic response and negative dB-values indicate increases (enhancement) in the acoustic response in presence of electrical stimulation.

Figure 4 shows an example of *Difference response* amplitude change as a function of acoustic stimulation frequency for electrical stimulation on electrode 1 vs. stimulation on electrode 2 (Subject CI04L). In this case, the pulse rate was constant at 421 pps. The *Difference response* amplitudes decreased for acoustic stimulus frequencies above 250 Hz, thereby providing evidence of peripheral physiologic electro-acoustic interactions. The *Difference response* amplitudes decreased up to 4 dB (dB re: 1 uV) at 250 Hz and about 2 dB above 250 Hz.

**Figure 5:**
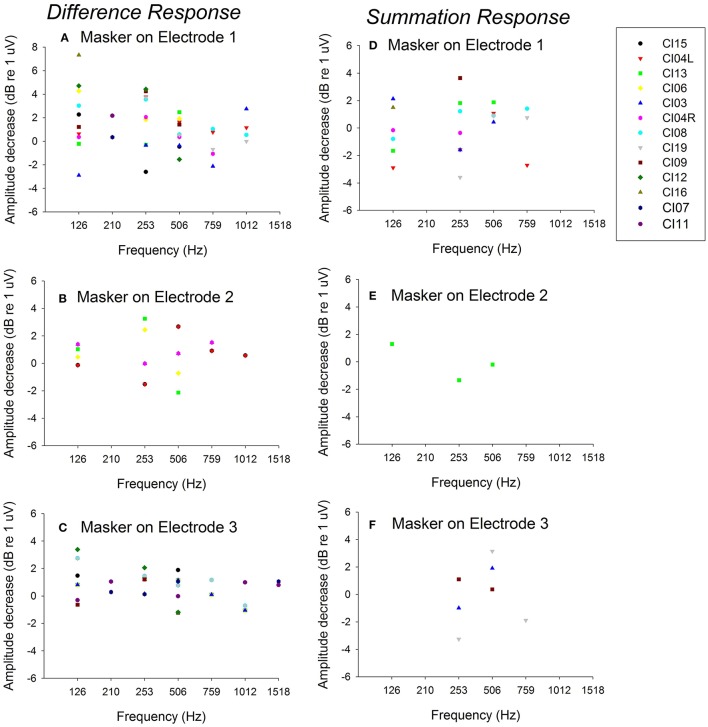
Comparison of *Derived acoustic* vs. *acoustic alone* for *Difference responses* (difference of alternating polarities) and *Summation responses* (summation of alternating polarities) for 13 ears across multiple frequencies. **(A–C)** show the data for *Difference responses* for electrical stimulation on electrodes 1–3; **(D–F)** show the data for *Summation responses* for electrical simulation on electrodes 1–3. The points above zero indicate decrease in acoustic response in presence of electrical stimulation (suppression) and points below zero indicate increase in acoustic response in presence of electrical stimulation (enhancement).

Figure 5 plots *Derived acoustic* vs. *acoustic-alone* responses for *Difference responses* across all 13 experimental ears for electrical stimulation on electrodes 1–3 for all test frequencies (Figures 5A–C). Figure 5 also shows the comparison of *acoustic alone* and *Derived acoustic* conditions for *Summation responses* for electrical stimulation on electrodes 1–3 for all test frequencies (Figures 5D–F). Data points above zero indicate decrease in acoustic response due to electrical stimulation (suppression) and points below zero indicate increase in acoustic response due to electrical stimulation (enhancement). The responses show significant electro-acoustic interactions for *Difference response* and non-significant electro-acoustic interactions for *Summation responses*. The difference between *Derived acoustic responses* and *Acoustic alone responses* was significant (two tailed *p* < 0.001, paired *t*-test, *n* = 41 for electrical stimulation on electrode 1; two tailed *p* < 0.001, paired *t*-test, *n* = 36 for electrical stimulation on electrode 3, two tailed *p* < 0.001, paired *t*-test, *n* = 20 for electrical stimulation on electrode 2) for *Difference responses*. The difference between *Derived acoustic responses* and *Acoustic alone responses* was not significant (two tailed *p* = 0.266, paired *t*-test, *n* = 21 for electrical stimulation on electrode 1; two tailed *p* = 0.89, paired *t*-test, *n* = 7 for electrical stimulation on electrode 3, two tailed *p* = 0.84, paired *t*-test, *n* = 3 for electrical stimulation on electrode 2) for *Summation responses*.

### Behavioral electro-acoustic interaction as a function of acoustic stimulus frequency (Experiment 2)

**Figure 6:**
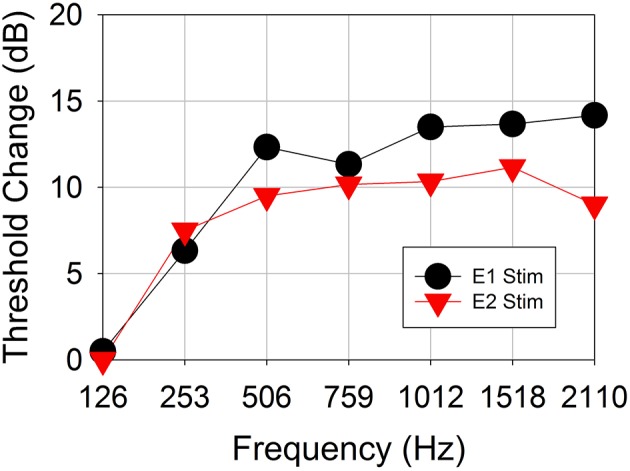
Behavioral threshold change vs. acoustic test frequency for a representative subject CI04L. An electrical masker of 421 pps was applied to either electrode 1 or electrode 3.

Figure 6 shows the changes in behavioral thresholds for one ear (Subject CI04L) in the presence of the 421-pps electrical masker. The observed variance between runs was 1 dB. The behavioral thresholds increased for test frequencies 250 Hz and above, with the greatest threshold shifts observed above 500 Hz. This subject did not show frequency selectivity with respect to threshold increase for stimulation on either electrode 1 or 2.

**Figure 7:**
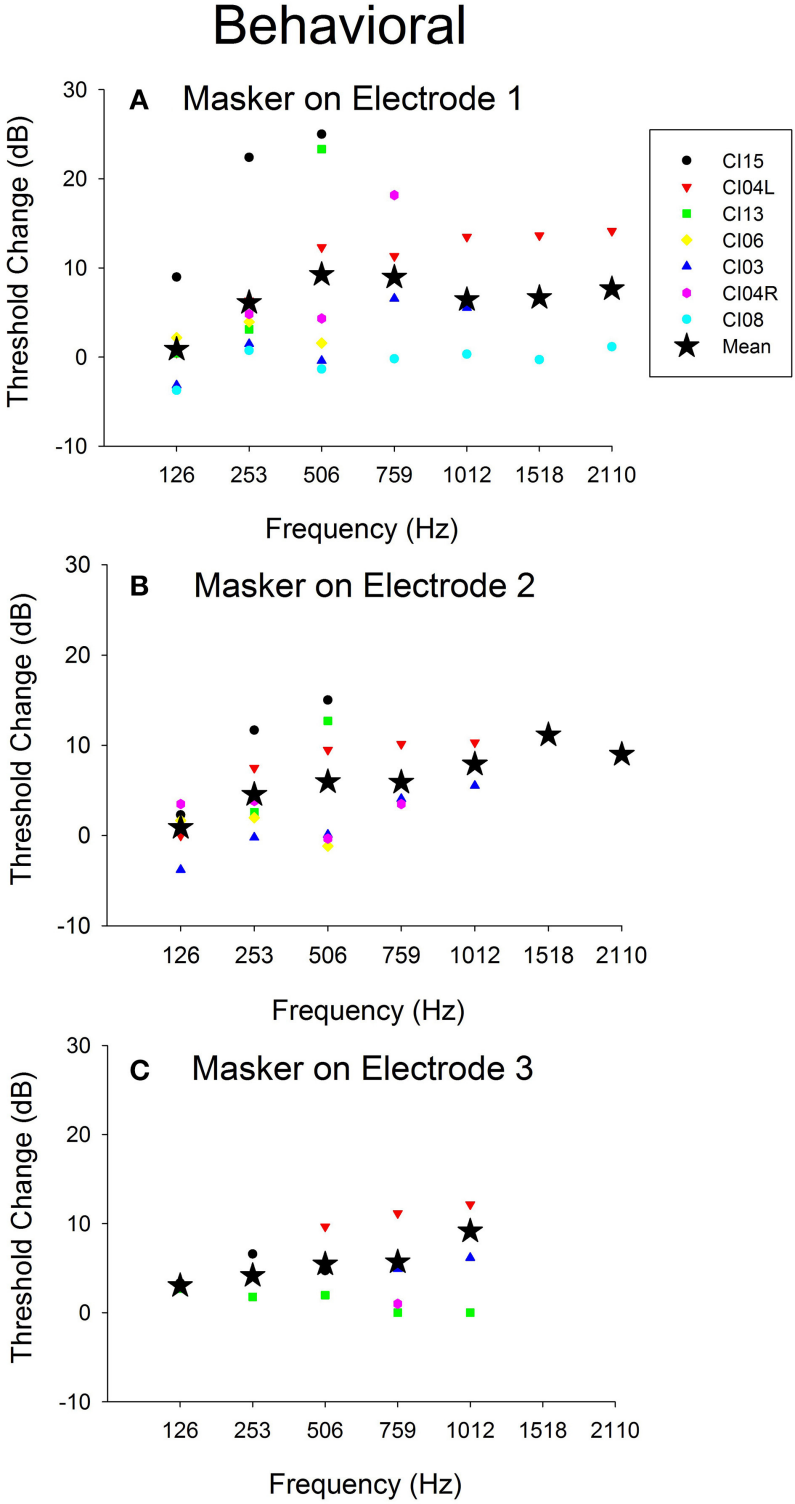
Behavioral threshold changes and *Difference response* amplitude changes vs. acoustic test frequency vs. electrical stimulation location. **(A–C)** Behavioral threshold changes observed with electrical stimulation masker on electrodes 1–3 across frequencies and across all subjects.

Figure 7 shows the effect of the 421-pps electrical masker across all audiometric test frequencies for all seven ears tested in Experiment 2. The data show mean and individual threshold changes observed across subjects. Each of the panels represents electrical stimulation on a different electrode. The mean data show the threshold selectivity of ~500–750 Hz for electrode 1 stimulation and ~1,500 Hz for electrode 2.

### Comparison of objective and behavioral electro-acoustic interaction

Figure 5 shows *Difference response* amplitude changes across audiometric frequencies in the presence of a 421-pps electrical masker for the same seven ears assessed in Experiment 2. Again, each of the panels represents electrical stimulation on a different electrode. The behavioral threshold shifts (Figure 7) varied by place of stimulation (i.e., by the electrode used for the masker) and the frequency of the acoustic probe stimulus. The behavioral thresholds show a peak around 500–750 Hz for electrical stimulation on electrode 1 and for higher frequencies for stimulation on electrodes 2 (~1,500 Hz). In contrast, the *Difference response* amplitude changes across electrodes do not show any clear peaks but do show greater shifts in amplitude for stimulation on electrode 1 than for stimulation on electrodes 2 or 3. This pattern suggests that apical stimulation results in greater physiologic electro-acoustic interactions than stimulation more basally. Notably, the objective electro-acoustic interactions estimated by *Difference response* amplitude changes (<8 dB change) were smaller than behavioral threshold changes (5–25 dB). There were no significant correlations observed between behavioral and objective electro-acoustic interactions (*p* > 0.05, *n* = 48, Pearson Correlation).

## Discussion

This study demonstrates the feasibility of measuring acoustic ECochG responses in the presence of electrical stimulation using the HiRes90K® Advantage cochlear implant. The fast-recovery amplifier enabled measurement of acoustic *Difference responses* and *Summation responses* for electrical pulse rates as high as 1,000 pps. Moreover, there were minimal or no residual electrical stimulation artifacts when using the technique described. Electro-acoustic interactions were observed in subset of subjects up to 4 dB of suppression in ECochG responses.

**Figure 8:**
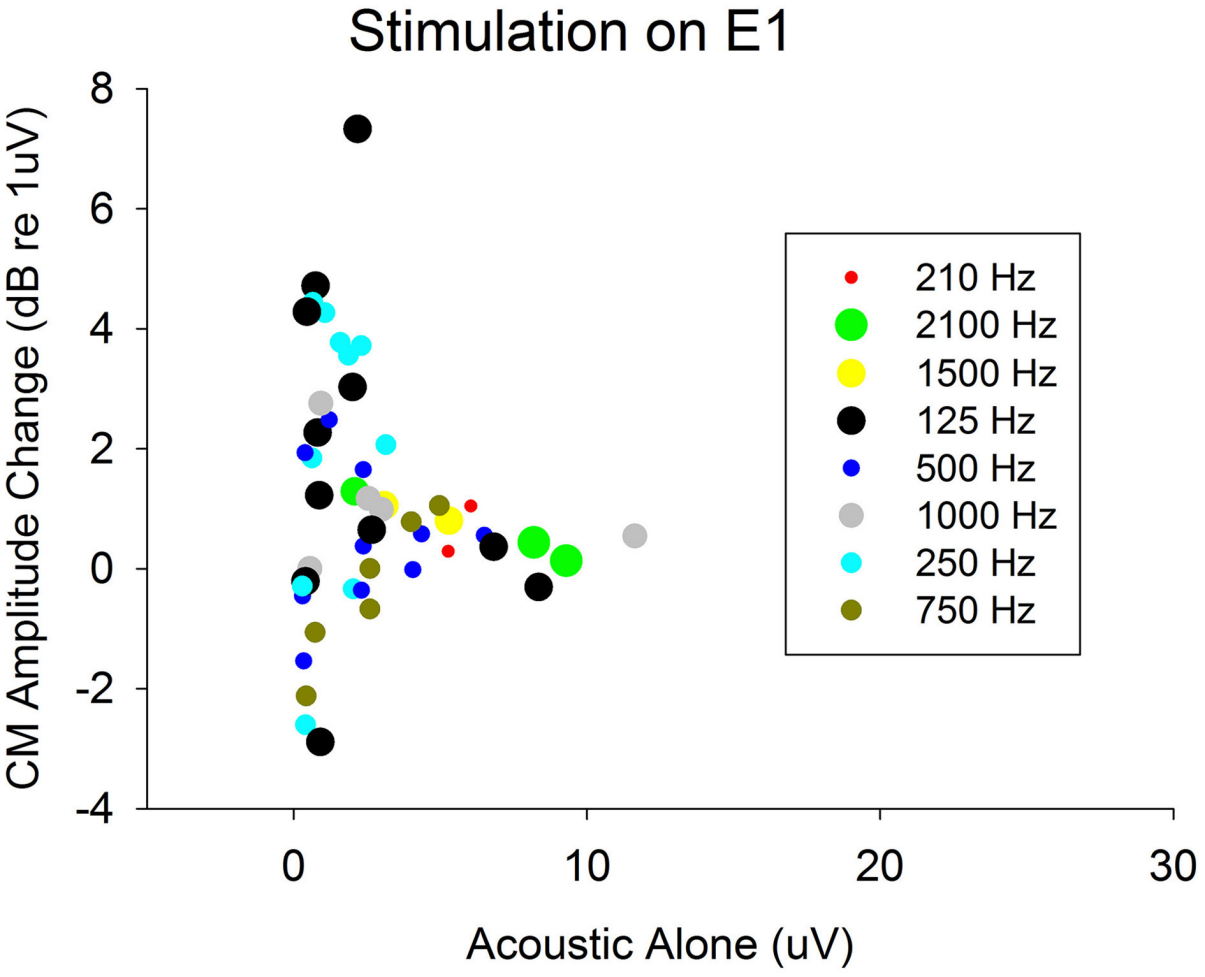
Decrease in acoustic responses due to electrical stimulation on electrode 1. The decrease in amplitudes (indicating presence of electro-acoustic interactions) were plotted with respect actual *acoustic alone* response amplitudes. This shows amount of electro-acoustic interactions observed dependent up on *acoustic alone* response amplitude (psychometric function at which the measures were obtained). The objective measures were obtained at either MCL or maximum of stimulus level of test system. This varied from soft level to MCL based on amount of residual hearing present.

Furthermore, this is the first study to demonstrate that ECochG can be used to evaluate electro-acoustic interactions in CI recipients with residual hearing. The degree of electro-acoustic interaction was dependent on location of the stimulation and recording electrode, as well as acoustic frequency (Figures 4, 5). Comparison of ECochG interactions and the effect of electrical stimulation on behavioral thresholds showed a general pattern of suppression of acoustic responses with electric stimulation. Quantitatively, the physiological measures showed less suppression than those observed behaviorally. For example, in the same subject, a 0–4 dB decrease in *Difference response* (Figure 4) corresponded to a 0–20 dB increase in behavioral threshold (Figure 6). One possible explanation is that the discrepancy between the two measures may be related to the difference in the point on the psychometric function at which the measures were obtained. ECochG measures were obtained with acoustic stimulation levels near MCL or maximum stimulus level of test system, whereas acoustic levels for the behavioral experiment were near threshold. The test stimulus level varied from soft level to MCL in different subjects based on their residual hearing (see Figure 1). Figure 8 shows the replot of the data from Figure 5A with X-axis changed to *Acoustic alone* response amplitude. This clearly shows that maximum interactions observed at smaller *acoustic alone* responses than at larger *acoustic alone* responses. The smaller *acoustic alone* response amplitudes indicate that test stimulus levels were at soft level and larger *acoustic alone* response amplitudes indicate that test stimulus levels were at MCL.

Stronks et al. (2010) reported a similar pattern where greater changes in acoustic CAPs in the presence of electric stimulation were observed near threshold compared to higher acoustic stimulus levels. Specifically, the amplitude changes observed at higher stimulation levels were around 3 dB, while amplitude changes were 10–20 dB near threshold. However, Stronks et al., study and other animal studies (Abbas et al., 2002) evaluated CAPs at higher acoustic frequencies than those used in this study. CAP techniques have limited applicability in CI recipients with low-frequency residual hearing where CAP responses cannot be measured. In contrast, *Difference responses* and *Summation responses* are measurable in these individuals at low frequencies (see Figure 3).

In summary, it is feasible to assess electro-acoustic interactions objectively in CI recipients with residual hearing. Further studies will explore stimulus-level-dependent electroacoustic interactions and whether these objective data can be used to guide fitting of EAS technology. Long-term, the goal is to be able to fit clinical EAS systems (1) without dependence on time-consuming psychometric methods and (2) in patients unable to undergo behavioral testing.

## Conclusions

It is feasible to record ECochG responses in the presence of electrical stimulation in HiRes90® Advantage CI recipients with residual hearing, thus providing a method for objectively assessing electro-acoustic interactions.

The HiRes90K® Advantage fast-recovery recording amplifier allows electro-acoustic interactions to be measured at high electrical stimulation rates with minimal stimulus artifacts.

Future studies are required to understand the relationship between behavioral and objective electro-acoustic interactions.

## Author contributions

Both authors contributed for conception and design, acquisition of data, or analysis and interpretation of data and drafting the article.

### Conflict of interest statement

The authors declare that the research was conducted in the absence of any commercial or financial relationships that could be construed as a potential conflict of interest.
